# Representation of Older Adults in the ACC/AHA/SCAI Guideline for Coronary Artery Revascularization

**DOI:** 10.1001/jamanetworkopen.2024.21547

**Published:** 2024-07-12

**Authors:** Yasser Jamil, Cosmas Sibindi, Dae Yong Park, Jennifer Frampton, Abdulla A. Damluji, Michael G. Nanna

**Affiliations:** 1 Department of Medicine, Yale School of Medicine, New Haven, Connecticut; 2 Department of Medicine, Cook County Health, Chicago, Illinois; 3 Section of Cardiovascular Medicine, Yale School of Medicine, New Haven, Connecticut; 4 Johns Hopkins University School of Medicine, Baltimore, Maryland; 5 Inova Center of Outcomes Research, Falls Church, Virginia

## Abstract

This cross-sectional study assesses the generalizability of the American College of Cardiology/American Heart Association/Society for Cardiovascular Angiography and Interventions (ACC/AHA/SCAI) guideline by examining the representation of older adults in studies cited in the guideline.

## Introduction

Older adults have the heaviest clinical burden across the coronary artery disease (CAD) spectrum.^1^ Despite this, they are less likely to be offered revascularization procedures that may improve symptoms, quality of life, and even cardiovascular mortality in select patients.^1,2^ Treatment gaps among older adults may be explained by underrepresentation in the literature underlying current guideline recommendations. We evaluated the representation of older adults in studies cited in the 2021 American College of Cardiology/American Heart Association/Society for Cardiovascular Angiography and Interventions (ACC/AHA/SCAI) Guideline for Coronary Artery Revascularization to assess the generalizability of the guidelines.^3^

## Methods

This cross-sectional study followed the Strengthening the Reporting of Observational Studies in Epidemiology (STROBE) reporting guideline. Institutional review board approval and informed patient consent were not required because this study used previously published, publicly available data without specific patient identifiers.

This cross-sectional study included the studies cited in the 2021 ACC/AHA/SCAI Guideline for Coronary Artery Revascularization.^3^ Screening and full-text review were conducted by 3 independent investigators (Y.J., C.S., D.Y.P.), and any disputes were resolved by discussion with the senior author (M.G.N.). The number of participants, mean age, and geriatric-centered outcomes (frailty, polypharmacy, falls, cognitive impairment, and functional independence) were documented. Representation of older adults was considered according to (1) active inclusion of older adults (aged 75 years or older or aged 80 years or older) in the methods or explicitly reporting subgroup analyses in these age groups; (2) having a first IQR or SD of 75 years or older or 80 years or older; (3) active exclusion based on age. Studies that did not report mean age, such as review articles or guidelines or those unrelated to cardiovascular conditions, were excluded. Subgroup analyses from the parent trial were considered if they specified the inclusion of older adults. Additional information can be found in eMethods in Supplement 1. The data analysis was descriptive and occurred from October 1981 to June 2021, and Excel (Microsoft) was used for calculations.

## Results

A total of 29 985 950 individuals from 540 studies were assessed (mean (SD) age, 66.0 [5.6] years), including 230 randomized clinical trials (RCTs) and 310 observational studies (Figure). This comprises 484 492 individuals in RCTs and 29 501 458 from observational studies, with mean (SD) ages of 63.4 (5.0) and 66.0 (6.0) years, respectively. When grouped according to an age cut-off, 4.4% of the included studies had a mean age of 75 years or older, and 1.5% had a mean age of 80 years or older. Moreover, 113 (20.9%) explicitly mentioned the inclusion of adults aged 75 years or older, of which 65 studies (12.0%) mentioned the inclusion of adults aged 80 years or older (Table). Of the studies that included older adults, the clinical presentation was relatively evenly distributed across CAD phenotypes. From the remaining studies that did not explicitly mention the inclusion of older adults, 146 studies (27.0%) had a mean (SD) age with the first IQR or SD inclusive of age 75 years or older, of which 29 studies (5.4%) had a mean age with IQR or SD of 80 years older. Conversely, 26 studies (4.8%) actively excluded adults aged 75 years or older, and 45 (8.3%) actively excluded adults aged 80 years or older. Lastly, only 11 studies (2.0%) reported geriatric-centered outcomes.

**Figure.**
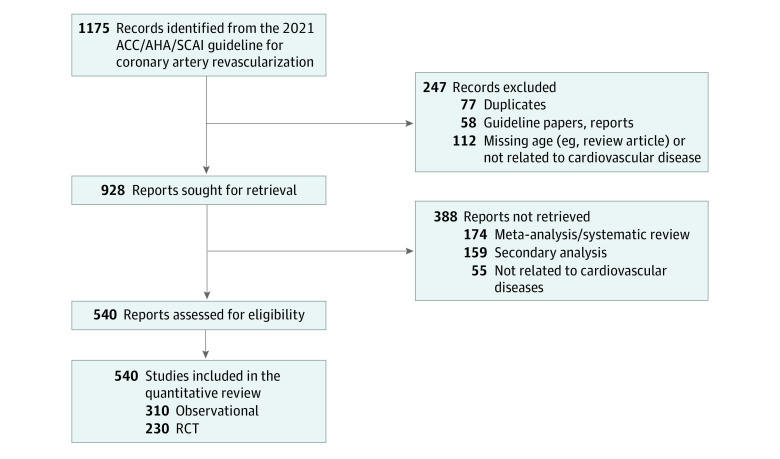
Cardiovascular Studies Selection Process The figure displays the cardiovascular studies selection process from the 2021 ACC/AHA/SCAI Guideline for Coronary Artery Revascularization. ACC indicates American College of Cardiology; AHA, American Heart Association; RCT, randomized clinical trial; SCAI, Society for Cardiovascular Angiography and Interventions.

**Table.** Representation of Older Adults in Observational Studies and RCTs Cited in the 2021 ACC/AHA/SCAI Guideline for Coronary Artery Revascularization

Description	Studies, No. (%)	
	With older adults aged ≥75 y	With older adults aged ≥80 y
No. of studies that included or excluded older adults	285	139
Inclusion based on methods or sub-group analysis	113/540 (20.9)	65/540 (12.0)
Inclusion based on IQR or SD	146/540 (27.0)	29/540 (5.4)
Active exclusion based on age	26/540 (4.8)	45/540 (8.3)
Classification of coronary disease in age inclusive studies, No. of studies	259	94
ST elevation myocardial infarction	93/259 (36)	33/94 (35)
Non-ST elevation myocardial infarction	88/259 (34)	35/94 (37)
Chronic coronary syndrome	78/259 (30)	26/94 (28)

Abbreviations: ACC, American College of Cardiology; AHA, American Heart Association; RCT, randomized clinical trial; SCAI, Society for Cardiovascular Angiography and Interventions.

## Discussion

These findings suggest that older adults with acute and chronic CAD were underrepresented in studies cited by the clinical practice guidelines for coronary artery revascularization. While the explicit exclusion of older adults has become less frequent than previously reported,^4^ less than 25% of the cited studies explicitly highlighted the inclusion of older adults, and few reported geriatric-centric outcomes. Given that older adults make up approximately one-third of individuals undergoing percutaneous coronary intervention for chronic CAD and a similar prevalence of individuals with acute coronary syndrome,^2,5^ these findings support the ongoing need for dedicated efforts to improve the representation of older adults in RCTs evaluating revascularization.

This study was limited by a lack of access to patient-level data, which precluded a precise estimate of the number of older adults included in the cited studies. However, as guideline recommendations play a crucial role for clinicians in making treatment decisions, the relative underrepresentation of older adults in the evidence base supporting these recommendations highlighted here may contribute to treatment gaps in older adults. Older adults frequently have unique physiological characteristics, increased procedural complexity, and a higher risk for adverse events following treatment.^1,6^ Additionally, older adults may have different treatment goals often not considered in revascularization studies, such as maintaining independence, function, cognition, and mobility. Thus, investigators and sponsors must prioritize the inclusion of older adults in revascularization studies to inform future guideline documents, reduce treatment heterogeneity, and assist clinicians in caring for a complex population of older adults with CAD.

## Notes

Supplement 1.
eMethods.

Supplement 2.
Data Sharing Statement
