# Supplementary material for: Representation of Older Adults in the ACC/AHA/SCAI Guideline for Coronary Artery Revascularization
Source: JAMA Netw Open. 2024 Jul 12;7(7):e2421547. doi: 10.1001/jamanetworkopen.2024.21547 (PMC11245718; doi:10.1001/jamanetworkopen.2024.21547)
Supplement: Supplement 1. — eMethods. [file jamanetwopen-e2421547-s001.pdf]

Supplemental Online Content

Jamil Y, Sibindi C, Park DY, Frampton J, Damluji AA, Nanna MG. Representation of older adults in the ACC/AHA/SCAI guideline for coronary artery revascularization. *JAMA Netw Open*. 2024;7(7):e2421547. doi:10.1001/jamanetworkopen.2024.21547

eMethods.

This supplemental material has been provided by the authors to give readers additional information about their work.

© 2024 Jamil Y et al. *JAMA Network Open.*

eMethods

Study type: Cross-sectional study. STROBE checklist was followed.

Search strategy and selection criteria

Representative search strategy: All references cited in the document: 2021 ACC/AHA/SCAI Guideline for Coronary Artery Revascularization: A Report of the American College of Cardiology/American Heart Association Joint Committee on Clinical Practice Guidelines. [1]

Data Extraction: Data was extracted on an Excel spreadsheet template that was agreed on before data extraction. All studies were eligible unless they met the exclusion criteria: missing age, review article, case report, meta-analysis/systematic review, or studies not related to cardiovascular disease.

Data sheet included: We extracted data for the following:
- Name of the first author and study, year of publication,
- type of study (observational or randomized controlled trial)
- The number of individuals included in the study,
- mean age,
- Exclusive inclusion of adults (≥75/80 years) if the full-text article mentioned inclusion based on age or if sub-group analysis was performed,
- Age inclusion of adults (≥75/80 years): if the mean/median age + first standard deviation or Interquartile range was above 75/80,
- Exclusion of adults ≥75/80 years
- Geriatric outcomes: frailty, dementia, cognitive impairment, falls, polypharmacy, dependence in daily living

Data analysis. The mean age, the total number of included individuals, the number of studies that included or excluded adults based on age cut-off, and the number of studies with geriatric outcomes were calculated on an Excel spreadsheet. This was a descriptive analysis.

Ethical review of study: Institutional review board approval and informed patient consent were not required because this study is a cross-sectional study of previously published, publicly available data without specific patient identifiers.

© 2024 Jamil Y et al. *JAMA Network Open.*

References:

1. Lawton JS, Tamis-Holland JE, Bangalore S, et al. 2021 ACC/AHA/SCAI Guideline for Coronary Artery Revascularization. *Journal of the American College of Cardiology*. 2022;79(2):e21-e129. doi:doi:10.1016/j.jacc.2021.09.006

© 2024 Jamil Y et al. *JAMA Network Open.*
