# Supplementary material for: Representation of Older Adults in the ACC/AHA/SCAI Guideline for Coronary Artery Revascularization
Source: JAMA Netw Open. 2024 Jul 12;7(7):e2421547. doi: 10.1001/jamanetworkopen.2024.21547 (PMC11245718; doi:10.1001/jamanetworkopen.2024.21547)
Supplement: Supplement 2. — Data Sharing Statement [file jamanetwopen-e2421547-s002.pdf]

Data Sharing Statement

Jamil. Representation of Older Adults in the ACC/AHA/SCAI Guideline for Coronary Artery Revascularization. *JAMA Netw Open*. Published July 12, 2024.
doi:10.1001/jamanetworkopen.2024.21547

Data
Data available: No
